# Glycolytic enzyme HK2 promotes PD-L1 expression and breast cancer cell immune evasion

**DOI:** 10.3389/fimmu.2023.1189953

**Published:** 2023-06-12

**Authors:** Jichun Lin, Wenshuo Fang, Zhuo Xiang, Qingqing Wang, Huapeng Cheng, Shimin Chen, Jing Fang, Jia Liu, Qiang Wang, Zhimin Lu, Leina Ma

**Affiliations:** ^1^ Department of Oncology, the Affiliated Hospital of Qingdao University, Qingdao, China; ^2^ Qingdao Cancer Institute, Qingdao, China; ^3^ School of Basic Medicine, Qingdao University, Qingdao, China; ^4^ Oncology Department, Shandong Second Provincial General Hospital, Jinan, China; ^5^ Department of Pharmacology, School of Pharmacy, Qingdao University, Qingdao, China; ^6^ Zhejiang Provincial Key Laboratory of Pancreatic Disease, The First Affiliated Hospital and Institute of Translational Medicine, Zhejiang University School of Medicine, Hangzhou, Zhejiang, China; ^7^ Cancer Center, Zhejiang University, Hangzhou, Zhejiang, China

**Keywords:** HK2, PD-L1, IκBα, NF-κB, immunotherapy, metabolism, breast cancer

## Abstract

Immune therapies targeting the PD-1/PD-L1 pathway have been employed in the treatment of breast cancer, which requires aerobic glycolysis to sustain breast cancer cells growth. However, whether PD-L1 expression is regulated by glycolysis in breast cancer cells remains to be further elucidated. Here, we demonstrate that glycolytic enzyme hexokinase 2 (HK2) plays a crucial role in upregulating PD-L1 expression. Under high glucose conditions, HK2 acts as a protein kinase and phosphorylates IκBα at T291 in breast cancer cells, leading to the rapid degradation of IκBα and activation of NF-κB, which enters the nucleus and promotes PD-L1 expression. Immunohistochemistry staining of human breast cancer specimens and bioinformatics analyses reveals a positive correlation between HK2 and PD-L1 expression levels, which are inversely correlated with immune cell infiltration and survival time of breast cancer patients. These findings uncover the intrinsic and instrumental connection between aerobic glycolysis and PD-L1 expression-mediated tumor cell immune evasion and underscore the potential to target the protein kinase activity of HK2 for breast cancer treatment.

## 1 Introduction

Breast cancer is commonly diagnosed cancer and is a leading cause of cancer-related deaths in females worldwide (1). Accumulated evidence has indicated that the immune system response is critical for the therapeutic efficacy and survival of breast cancer patients. In addition, breast cancer cells exhibit immune evasion capabilities (2, 3). Tumor cell membrane protein programmed cell death ligand1 (PD-L1, also known as B7-H1) binds to the receptor protein programmed cell death 1 (PD-1) on the surface of T lymphocyte cells, resulting in the blockage of T cell proliferation, cytokine production, and the inhibition of the immune response (4–6). PD-L1 expression is often upregulated in breast cancer cells and plays a role in immune evasion (7, 8). A study on breast cancer patients showed that the abnormal expression of PD-L1 was closely related to the reduction of overall survival rate and poor prognosis (9). PD-1/PD-L1 immune checkpoint inhibitors have been used in various cancer treatments, including clinical trials in breast carcinoma. However, a portion of patients did not respond to the immunotherapy (2, 10). Therefore, further research on the regulation of PD-L1 expression in breast cancer cells will shed light on the mechanism underlying breast cancer cell immune evasion and help increase immune checkpoint therapy’s clinical effectiveness.

Nuclear factor kappa-light-chain-enhancer of activated B cells (NF-κB) is a nuclear transcription factor highly expressed in breast cancer tissues (11, 12). In unstimulated cells, NF-κB composed of Rel A (p65)/p50 dimers is bound by IκBα protein and sequestrated in the cytoplasm. In response to cytokine stimulation, IκBα undergoes rapid ubiquitylation-mediated proteasome degradation that releases the bound, cytoplasmic NF-κB dimers (13). Then, NF-κB enters the nucleus and promotes PD-L1 transcription (14, 15). NF-κB can be regulated by hexokinase (HK) in glioblastoma cells (16). HK is a rate-limiting enzyme in aerobic glycolysis, which converts glucose to the metabolic intermediate glucose-6-phosphate (G-6-P) (17). Four isotypes of the HK family are founded in mammals: HK1, HK2, HK3, and HK4 (18, 19). HK2 binds to mitochondrial outer membrane voltage-dependent anion channel 1 (VDAC1) protein (20, 21), which enables HK2 to utilize ATP produced by mitochondria for glycolysis. High glycolysis-produced large amount of G-6-P disassociates HK2 from the mitochondria by a feedback-regulated mechanism (22). The expression of HK2, which can be induced by erbB2/Neu (23), was significantly increased in breast cancer specimens compared to normal tissue (24). HK2 deletion inhibited breast cancer metastasis (25). HK2 not only has the function of a glycolytic enzyme but also has non-metabolic functions (16, 26, 27). A recent study demonstrated that HK2 in glioblastoma cells acts as a protein kinase and phosphorylates IκBα, resulting in IκBα degradation and NF-κB activation for PD-L1 transcription (16). However, the relationship between HK2 and immunoregulation in breast cancer remains unclear.

In this study, we demonstrated that aerobic glycolysis induces PD-L1 expression in an HK2-dependent manner. HK2 phosphorylates IκBα at T291, resulting in IκBα rapid degradation and NF-κB activation, resulting in enhanced PD-L1 transcription and breast cancer cell immune evasion.

## 2 Materials and methods

### 2.1 Materials

Rabbit antibodies that recognize human HK2 (Cat#ab209847; RRID: AB2904621) and p65 (Cat#ab32536; RRID: AB776751) were obtained from Abcam (Shanghai, China). Rabbit antibodies against PD-L1 (Cat#ab13684; RRID: AB2687655) and α-tubulin (Cat#ab2125; RRID: AB2619646) and mouse antibody against IκBα (Cat#ab4814; RRID: AB390781) were purchased from Cell Signaling Technology. Rabbit antibodies against Flag (Cat#20543-1-AP; RRID: AB11232216) and histone H3 (Cat#ab17168; RRID: AB2716755) were purchased from Proteintech (Wuhan, China). Rabbit polyclonal anti-IκBα pT291 from Signalway Biotechnology (Pearland. TX). Goat anti-rabbit IgG (H+L) secondary antibody (Cat#A-11008; RRID: AB-143165) was obtained from Invitrogen. G-6-P (Cat#D9434) was purchased from Sigma (Shanghai, China). Glucose (Cat#A501991) was obtained from Sangon Biotech (Shanghai, China). CHX (HY-12320) was purchased from MedChemExpress (Shanghai, China). Lipofectamine 2000 (L3000015) transfection reagents and Blasticidin (Cat#R21001) were obtained from Thermo Fisher Scientific (Waltham, MA).

### 2.2 Cell culture and cell transfection

Human breast cancer MCF-7 (RRID: CVCL 0031), BT-549 (RRID: CVCL 1092), SK-BR-3 (RRID: CVCL 0033), and human embryonic kidney 293T (RRID: CVCL LF52) cells were purchased from ATCC and maintained in Dulbecco’s modified Eagle’s medium (DMEM) or McCoy’s 5A medium supplemented with 10% fetal bovine serum (FBS) and 1% penicillin/streptomycin at 37°C with 5% CO_2_. The transfection using Lipofectamine2000 reagent (Invitrogen) was performed as previously described (28). For G-6-P treatment, 1M G-6-P was mixed with 5 μl Lipofectamine2000 in OPTI-MEM for 30 minutes at room temperature and supplemented into the culture medium in a 6-well plate.

### 2.3 Subcellular fractionation

Nuclear and cytosolic fractions were prepared as previously described (29). Briefly, Flag-HK2 or vector was transfected into MCF-7 cells with Lipofectamine2000 reagent (Invitrogen). 48 h later, cells were collected and suspended in 300 μl Buffer A (10 mM HEPES, 10 mM KCL, 0.1 mM EDTA,0.1 mM EGTA, 0.15% NP-40, protease inhibitors), shaken by hand, and placed on ice for 10 min, 13000 rpm at 4°C for 30 seconds, and the supernatant is the cytoplasm. Then, the precipitate was suspended with 700 μl Buffer A, left for 3min, 13000 rpm for 30 seconds at 4°C to clean the nuclear components. Repeat the above steps 2 times to wash the remaining pulp components from the core. Discard the supernatant and add 70 μl CST lysis, 25% ultrasonic for 6 times, centrifuged at 13000 rpm for 20 min at 4°C. The supernatant is the nuclear component.

### 2.4 Quantitative PCR

Quantitative PCR analyses were performed as described previously (30). Total RNA was extracted from cells using TRIzol reagent and reverse transcribed with Maxima Reverse Transcriptase according to the manufacturer’s instructions. Quantitative PCR analysis was carried out using a 7500 Real-Time PCR system (Applied Biosystems) with an SYBR Premix ExTaq kit (Bimake). The relative expression was determined using the ΔΔCT method of normalization. The following primers were used for quantitative PCR, Human CD274 forward: 5’-CTGCACTTTTAGGAGATTAGATC-3’; Human CD274 reverse: 5’-CTACACCAAGGCATAATAAGATG-3’; Human β-actin forward: 5’-TGGCACCCAGCACAATGAA-3’; Human β-actin reverse: 5’-CTAAGTCATAGTCCGCCTAGAAGCA-3’.

### 2.5 Western blot analysis

Total proteins were extracted with CST lysis buffer containing protease and phosphatase inhibitors. The protein concentration was determined using a Bradford reagent kit (Thermo Fisher Scientific), and proteins were separated by SDS-PAGE and transferred to PVDF membranes. Membranes were blocked with 5% milk for 1 hour and then incubated with primary antibody at 4°C overnight. Membranes were washed with Tris-buffered saline containing Tween-20, incubated with secondary antibodies, and developed with an enhanced chemiluminescence kit.

### 2.6 Flow cytometry analysis

Flow cytometry analysis was performed as described previously (31). Cells were fixed with 4% paraformaldehyde for 15 minutes at room temperature and then were washed with PBS. An anti-PD-L1 antibody was added to the cells for 1 hour at room temperature. The cells were washed with PBS three times. A fluorescence antibody was added to the cells for 30 minutes at room temperature. After incubation, the cells were washed with PBS and detected by a Beckman cytometer.

### 2.7 Immunoprecipitation analysis

Immunoprecipitation analysis using antibodies as described previously (32). Briefly, cells were collected and lysed in CST lysis buffer (20 mM Tris-HCl [pH7.5], 150 mM NaCl, 1 mM Na_2_EDTA.2H_2_O, 1 mM EGTA, 1% TritonX-100 and 2.5 mM Na_4_P_2_O_7_) containing protease inhibitor cocktail (Bimake) and phosphatase inhibitor cocktails (Bimake). For coimmunoprecipitation, the cell lysate supernatant was mixed with indicated antibodies overnight at 4°C and incubated with 30 μl protein A/G agarose beads for 3 hours at 4°C on a rocking platform and then washed the beads 3 times with NETN buffer (20 mM Tris-HCl [pH8.0], 100 mM NaCl, 1 mM EDTA, 0.5% NP-40) and boiled with 50 μl of 2×SDS loading buffer for 10 min. Finally, the obtained proteins were subject to Western blotting.

### 2.8 Lentiviral generation and infection

Lentiviral constructs expression shControl and shHK2 were co-transfected into HEK293T cells with package plasmids with PEI (Invitrogen) as described previously (33). Lentivirus was collected 72 hours after transfection and was filtered by a 0.45 μm filter membrane. The filter lentivirus was infected with MCF-7 using 10 μg/ml polybrene. Screening stable expression cells by Blasticidin.

### 2.9 Patients and tissue samples

We retrospectively collected 220 human breast carcinoma specimens from Shandong Second Provincial General Hospital (Jinan, China), and obtained clinical data by reviewing the patients’ medical histories.

### 2.10 Ethics statement

The studies involving human breast cancer specimens and the database were approved by the institutional research ethics committee of the Oncology Department, Shandong Second Provincial General Hospital. All patients involved in the study were conducted strictly with the national ethical policy. Informed consent was obtained from all the patients whose tissue samples were allowed to be used for scientific research, and patient privacy was protected.

### 2.11 Immunohistochemical analysis

IHC staining was performed using the VECTASTAIN ABC kit (Vector Laboratories) according to the manufacturer’s instructions. Human breast cancer tissues were stained with antibodies HK2 (dilution 1:500), PD-L1 (dilution 1:400), IκBα pT291 (dilution 1:50) or nonspecific IgG (as a negative control). We quantitatively scored the sections based on the percentage of positive cells and the intensity of staining of the sections (34). The staining intensity is scored as follows: 0, no signal; 1, weak; 2, moderate; and 3, strong. The IHC scores were assessed by independent pathologists. We then multiply the intensity and percentage of positive cells to obtain a total score.

### 2.12 TIMER database analysis

TIMER (http://timer.cistrome.org/) is an estimating immune cell infiltration database and provides comprehensive analysis and visualization functions of tumor infiltrating immune cells which uses data from TCGA (35–37). In the study, we examined the correlation between HK2 mRNA levels and CD274 mRNA levels. Then, we examined tumor-infiltrating CD4^+^ T cells through TIMER algorithm and tumor-infiltrating CD8^+^ T cells through CIBERSORT algorithm in TIMER2.0 database. Spearman’s rho value was used to evaluate the degree of their correlation. HK2 expression and breast cancer patient survival analysis was tested using the Kaplan-Meier Plotter (https://kmplot.com/analysis/) (38, 39), which searched for breast cancer cohorts in NCBI Gene Expression Omnibus (https://www.ncbi.nlm.nih.gov/geo/) and in the Genomic Data Commons Data Portal (https://portal.gdc.cancer.gov/).

## 3 Results

### 3.1 High glucose enhances PD-L1 expression in an HK2-dependent manner

**Figure 1:**
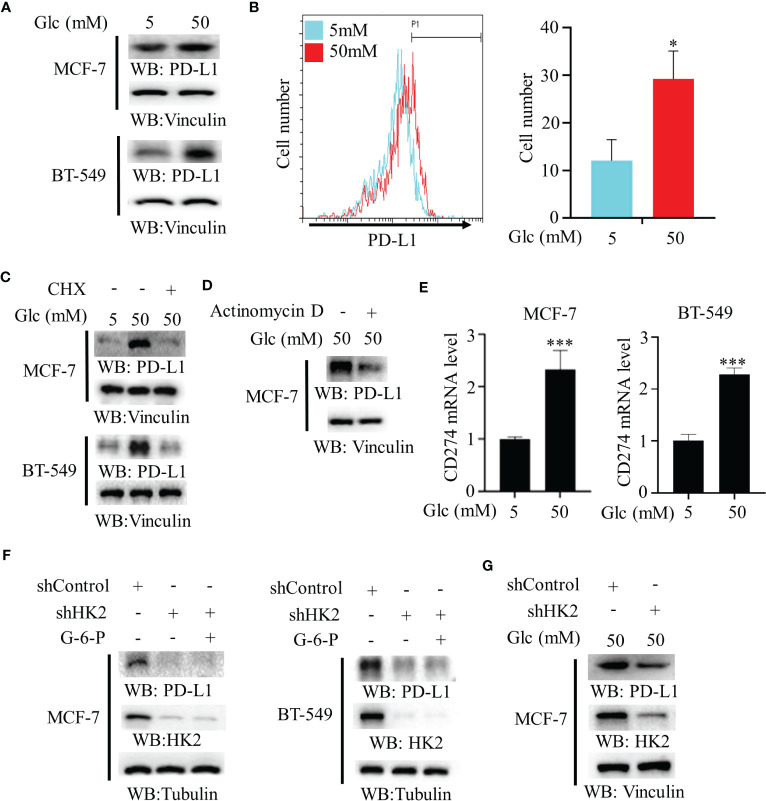
High glucose enhances PD-L1 expression in an HK2-dependent manner. **(A)**, MCF-7 and BT-549 cells were treated with the indicated glucose concentrations for 24 h. Immunoblotting analyses were performed with the indicated antibodies. **(B)**, MCF-7 cells were treated with low (5 mM) or high glucose (50 mM) for 24 h. Flow cytometry analyses were performed. *p < 0.05. **(C)**, MCF-7 and BT-549 cells were treated with the indicated glucose concentrations for 24 h in the presence or absence of cycloheximide (CHX) (100 μg/ml). Immunoblotting analyses were performed with the indicated antibodies. **(D)**, MCF-7 cells were cultured with high glucose (50 mM) for 24 h with or without pretreatment with actinomycin D (1 μg/ml). **(E)**, Real-time PCR analyses of *CD274* mRNA in MCF-7 cells and BT-549 cells cultured with the indicated glucose concentrations for 24 h. Data are the means ± SD of 3 independent experiments. ***p < 0.001. **(F)**, MCF-7 and BT-549 cells stably expressing a control shRNA or HK2 shRNA were treated with or without G-6-P for 12 h. Immunoblotting was performed with the indicated antibodies. **(G)**, MCF-7 cells stably expressing a control shRNA or HK2 shRNA were cultured in medium containing high glucose (50 mM). Immunoblotting analyses were performed with the indicated antibodies. *p<0.05.

To determine whether changes of glucose level modulate PD-L1 expression in breast cancer cells, we treated MCF-7 and BT-549 cells with different concentrations of glucose. We found that a high glucose concentration increased PD-L1 expression (**Figure 1A**). In addition, flow cytometry analyses revealed that high glucose concentration enhanced PD-L1 expression on the surface of MCF-7 cells (**Figure 1B**). This increase was decreased by treatment with both protein synthesis inhibitor cycloheximide (CHX) (**Figure 1C**) and transcription inhibitor actinomycin D (**Figure 1D**), suggesting extracellular glucose levels regulate PD-L1 at both transcriptional and posttranslational levels. Consistent with this finding, quantitative PCR analyses showed that high glucose treatment increased mRNA expression of the *CD274* gene (encoding PD-L1) in MCF-7 and BT-549 cells (**Figure 1E**). Notably, depletion of HK2 by expression of its shRNA in MCF-7 and BT-549 cells reduced PD-L1 expression, and this reduction was not rescued by supplementation with HK2 product G-6-P (**Figure 1F**), suggesting that glycolytic reactions downstream of HK2 are not involved in the regulation of PD-L1 expression. Consistently, HK2 depletion decreased PD-L1 expression in MCF-7 cells under high glucose conditions (**Figure 1G**). These results indicated that high glucose upregulates PD-L1 expression in an HK2-dependent manner.

### 3.2 HK2-mediated IκBα phosphorylation reduces IκBα expression

**Figure 2:**
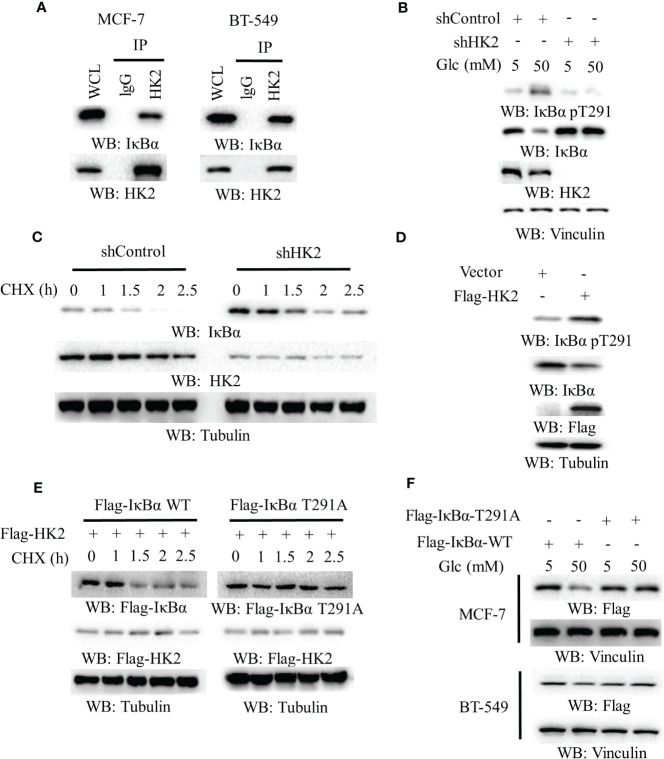
HK2-mediated IκBα phosphorylation reduces IκBα expression. **(A)**, MCF-7 and BT-549 cells were analyzed by immunoprecipitation and immunoblotting analyses with the indicated antibodies. **(B)**, MCF-7 cells stably expressing a control shRNA or HK2 shRNA were cultured in medium containing the indicated concentrations of glucose for 24 h. Immunoblotting analyses were performed with the indicated antibodies. **(C)**, MCF-7 cells with or without HK2 shRNA were treated with cycloheximide (CHX) (100 μg/ml) and harvested at the indicated periods of time. Immunoblotting analyses were performed with the indicated antibodies. **(D)**, A control vector or a vector expression Flag-HK2 was transfected into MCF-7 cells. Immunoblotting analyses were performed with the indicated antibodies. **(E)**, MCF-7 cells expressing Flag-HK2, WT Flag-IκBα or Flag-IκBα T291A were treated with CHX (100 μg/ml) for the indicated periods of time. Immunoblotting analyses were performed with the indicated antibodies. **(F)**, WT Flag-IκBα or Flag-IκBα T291A was expressed in MCF-7 and BT-549 cells. The cells were cultured with the indicated concentrations of glucose for 24 h.

HK2 phosphorylates IκBα T291 and promotes IκBα degradation in glioblastoma cells (16). To define the mechanism underlying HK2-upregulated PD-L1 expression in breast cancer cells, we performed co-immunoprecipitation analyses and showed that endogenous HK2 interacted with endogenous IκBα in MCF-7 and BT-549 cells (**Figure 2A**). In addition, high glucose-induced IκBα T291 phosphorylation and decreased IκBα expression. Notably, this change was abrogated by HK2 depletion (**Figure 2B**), which prolonged the half-life of IκBα (**Figure 2C**). Consistently, Flag-HK2 overexpression considerably enhanced IκBα T291 phosphorylation and reduced IκBα expression (**Figure 2D**) and decreased the half-life of wild-type (WT) IκBα compared to that of IκBα T291A (**Figure 2E**). In contrast to WT Flag-IκBα, Flag-IκBα T291A displayed resistance to degradation in MCF-7 and BT-549 cells upon high glucose treatment (**Figure 2F**). These results indicated that HK2 phosphorylates IκBα T291 phosphorylation and decreases IκBα expression under high glucose conditions.

### 3.3 Overexpression of HK2 induces nuclear translocation of p65 and CD274 transcription

**Figure 3:**
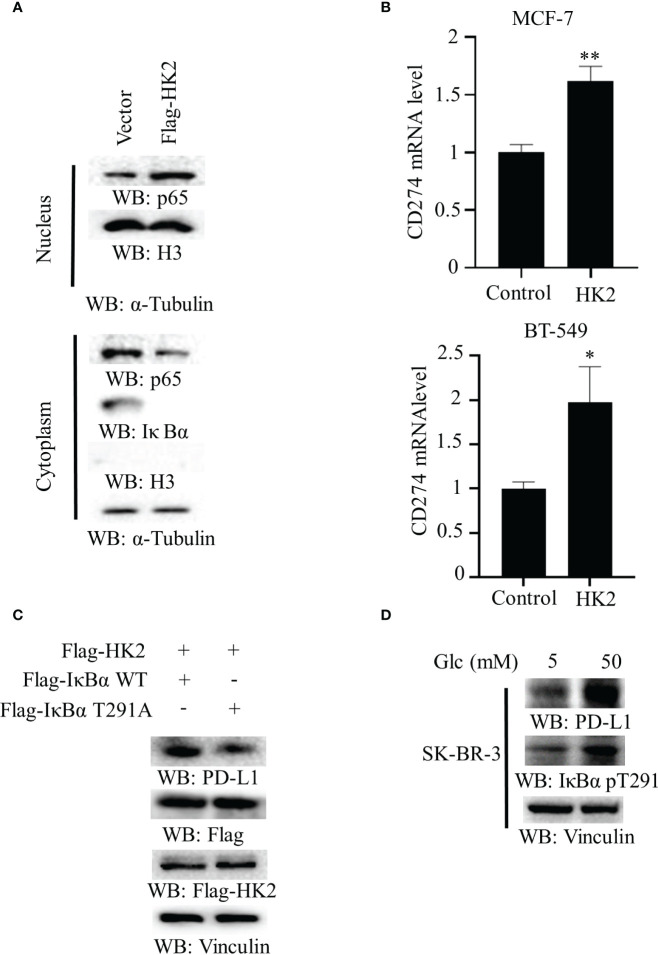
Overexpression of HK2 induces nuclear translocation of p65 and *CD274* transcription. **(A)**, Cytoplasmic and nuclear fractions of MCF-7 cells with or without expressing Flag-HK2 were analyzed by immunoblotting analyses with the indicated antibodies. **(B)**, MCF-7 and BT-549 cells were transfected with a control vector or Flag-HK2 for 48 hours. A real-time PCR analysis was performed. Data are the means ± SD of 3 independent experiments. **p < 0.01, *p < 0.05. **(C)**, Flag-HK2, WT Flag-IκBα or Flag-IκBα T291A was expressed in MCF-7 cells. Immunoblotting analyses were performed with the indicated antibodies. **(D)**, SK-BR-3 cells were cultured in medium containing low (5 mM) or high glucose (50 mM) for 24 h. Immunoblotting analyses were performed with the indicated antibodies.

To determine whether aerobic glycolysis regulates the NF-κB in breast cancer cells, we overexpressed Flag-HK2 in MCF-7 cells. We found that Flag-HK2 expression promoted the nuclear translocation of p65 with a corresponding decrease of IκBα expression in the cytosol (**Figure 3A**). In addition, HK2 overexpression considerably elevated the mRNA level of CD274 in both MCF-7 and BT-549 cells (**Figure 3B**) and increased expression of WT Flag-IκBα to a higher level than that of IκBα T291A (**Figure 3C**). Notably, high glucose conditions also enhanced IκBα T291 phosphorylation and PD-L1 expression in HER2-positive SK-BR-3 breast cancer cells (**Figure 3D**), suggesting that HK2-regulated PD-L1 expression is independent of HER2 expression. These results suggested that HK2-mediated IκBα T291 phosphorylation promotes nuclear translocation of p65 and PD-L1 expression.

### 3.4 HK2 expression is positively correlated with CD274 expression and negatively associated with CD8^+^ T cell infiltration and survival time of breast cancer patients

**Figure 4:**
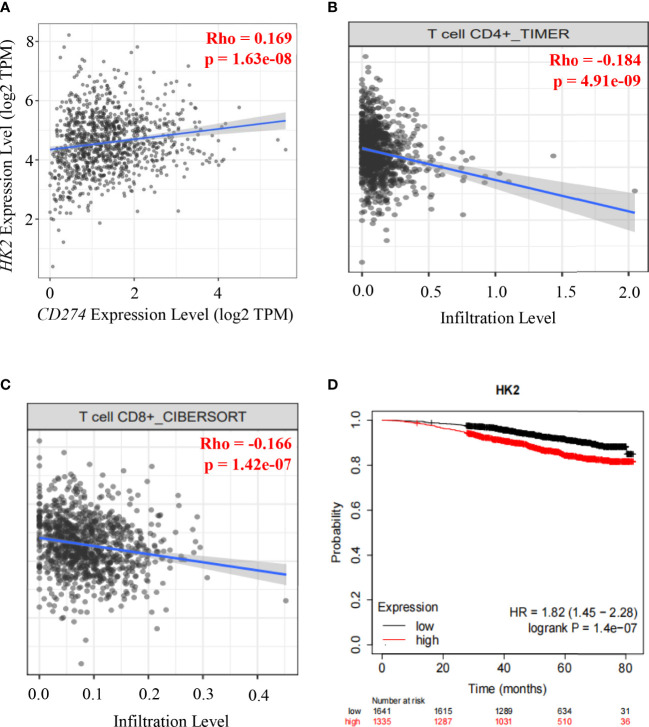
HK2 expression is positively correlated with CD274 expression and negatively associated with CD8^+^ T cell infiltration and survival time of breast cancer patients.**(A)**, Correlative expression of *CD274* mRNA with HK2 mRNA expression in the TCGA cohort of BRCA samples (n = 1100) was analyzed. Spearman’s rho value is presented for correlations. **(B)**, The correlation between HK2 mRNA expression levels and the infiltrating levels of CD4^+^ T cells was analyzed by TIMER algorithm in the TIMER2.0 database in breast cancer patients specimens (n=1100). Spearman’s rho value is presented for correlations. **(C)**, The correlation between HK2 mRNA expression levels and the infiltrating levels of CD8^+^ T cells was analyzed through CIBERSORT algorithm in TIMER2.0 database in breast cancer patients specimens (n=1100). Spearman’s rho value is presented for correlations. **(D)**, The association between HK2 mRNA expression levels and breast cancer patient survival was analyzed using the Kaplan Meier plotter database.

To determine whether HK2 expression is correlated with PD-L1 expression in human breast cancer specimens, we analyzed 1100 breast cancer cases in The Cancer Genome Atlas (TCGA) database. We revealed that HK2 mRNA levels were positively associated with CD274 mRNA levels (correlation: 0.169, p=1.63e-08) (**Figure 4A**). Analyses of the associations between HK2 expression and immune cells infiltration using the TIMER2.0 database (40), which showed that HK2 mRNA levels in breast cancer specimens were inversely correlated with the infiltration of CD4^+^ T cells (correlation: 0.184, p=4.91e-08) (**Figure 4B**) and CD8^+^ T cells (correlation: 0.166, p=1.42e-07) (**Figure 4C**) through TIMER algorithm and CIBERSORT algorithm analyses, respectively. In addition, analyses of the association between HK2 expression and breast cancer patient survival using the Kaplan Meier plotter database (https://kmplot.com) revealed that HK2 expression levels were inversely correlated with the survival time of breast cancer patients (**Figure 4D**). These results indicated that HK2 expression is positively correlated with CD274 expression and negatively associated with CD8^+^ T cell infiltration and survival time of breast cancer patients.

### 3.5 HK2 expression is positively correlated with IκBα T291 phosphorylation and PD-L1 expression in human breast cancer specimens

**Figure 5:**
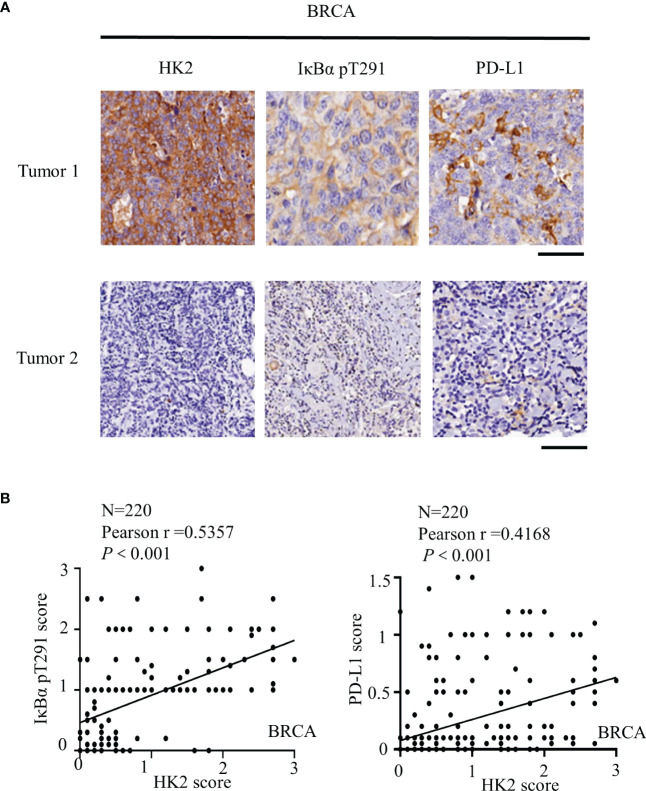
HK2 expression is positively correlated with IκBα T291 phosphorylation and PD-L1 expression in human breast cancer specimens. **(A)**, IHC analyses 220 human breast cancer specimens with HK2, PD-L1 and IκBα T291 antibodies. Two representative tumor IHC staining images were shown. Scale bars, 100μM. **(B)**, IHC staining was scored, and the correlations between the expression levels of HK2, PD-L1, and IκBα T291 phosphorylation were analyzed by Pearson correlation test. Note that some of the dots on the graphs are overlapped.

To further determine the clinical significance of HK2-mediated IκBα T291 phosphorylation, thereby promoting the expression of PD-L1 in breast cancer patients, we performed immunohistochemistry (IHC) analyses of 220 breast cancer specimens with a specificity-validated anti-IκBα T291antibody and antibodies against HK2 and PD-L1 (16). We analyzed the correlation between HK2 expression and clinicopathological characteristics. We found a positive correlation of HK2 expression levels with larger tumor sizes, progesterone receptor (PR)-negative expression, and higher Ki67 levels (**Table 1**). In addition, IHC staining showed that HK2 expression levels were positively correlated with levels of IκBα T291 phosphorylation and PD-L1 expression (**Figure 5A**). Statistical analysis showed that these correlations were significant (**Figure 5B**). These results support the role of HK2-mediated IκBα T291 phosphorylation in upregulated PD-L1 expression in breast cancer specimens.

**Table 1:** The Correlation between HK2 Expression and Clinicopathological Characteristics in Breast Cancer Patients (n=220 cases).

Characteristic	Number (%)	HK2 expression		p value
Total	220	Positive (103, 46.82%)	Negative (117, 53.18%)	
Age, years				
<50	64 (29.09%)	24 (10.91%)	40 (18.18%)	0.076
≥50	156 (70.91%)	79 (35.91%)	77 (35.00%)	
Tumor size, cm				
≤2	62 (28.18%)	20 (9.09%)	42 (19.09%)	0.021
2~5	123 (55.91%)	63 (28.64%)	60 (27.27%)	
≥5	35 (15.91%)	20 (9.09%)	15 (6.82%)	
Histological grades				
I	23(10.45%)	7 (3.18%)	16 (7.27%)	0.151
II	127 (57.73%)	70 (31.82%)	67 (30.45%)	
III	70 (31.82%)	26(11.82%)	34 (15.45%)	
Lymph node status				
0	138 (62.73%)	60 (27.27%)	78 (35.45%)	0.436
1-3	42 (19.09%)	22 (10.00%)	20 (9.09%)	
≥4	40 (18.18%)	21 (9.55%)	19 (8.64%)	
ER				
Positive	156 (70.91%)	68 (30.91%)	88 (40.00%)	0.134
Negative	64 (29.09%)	35 (15.91%)	29 (13.18%)	
PR				
Positive	144 (65.45%)	60 (27.27%)	84 (38.18%)	0.035
Negative	76 (34.55%)	43 (19.55%)	33 (15.00%)	
HER2				
Positive	46 (20.91%)	26 (11.82%)	20 (9.09%)	0.138
Negative	174 (79.09%)	77 (35.00%)	97(44.09%)	
Ki67				
≥30%	110 (50.00%)	62 (28.18%)	48 (21.82%)	0.006
<30%	110 (50.00%)	42 (18.64%)	69 (31.36%)	

ER, estrogen receptor; PR, progesterone receptor; HER-2, human epidermal growth factor receptor 2. Two-sides Chi-Square tests.

## 4 Discussion

**Figure 6:**
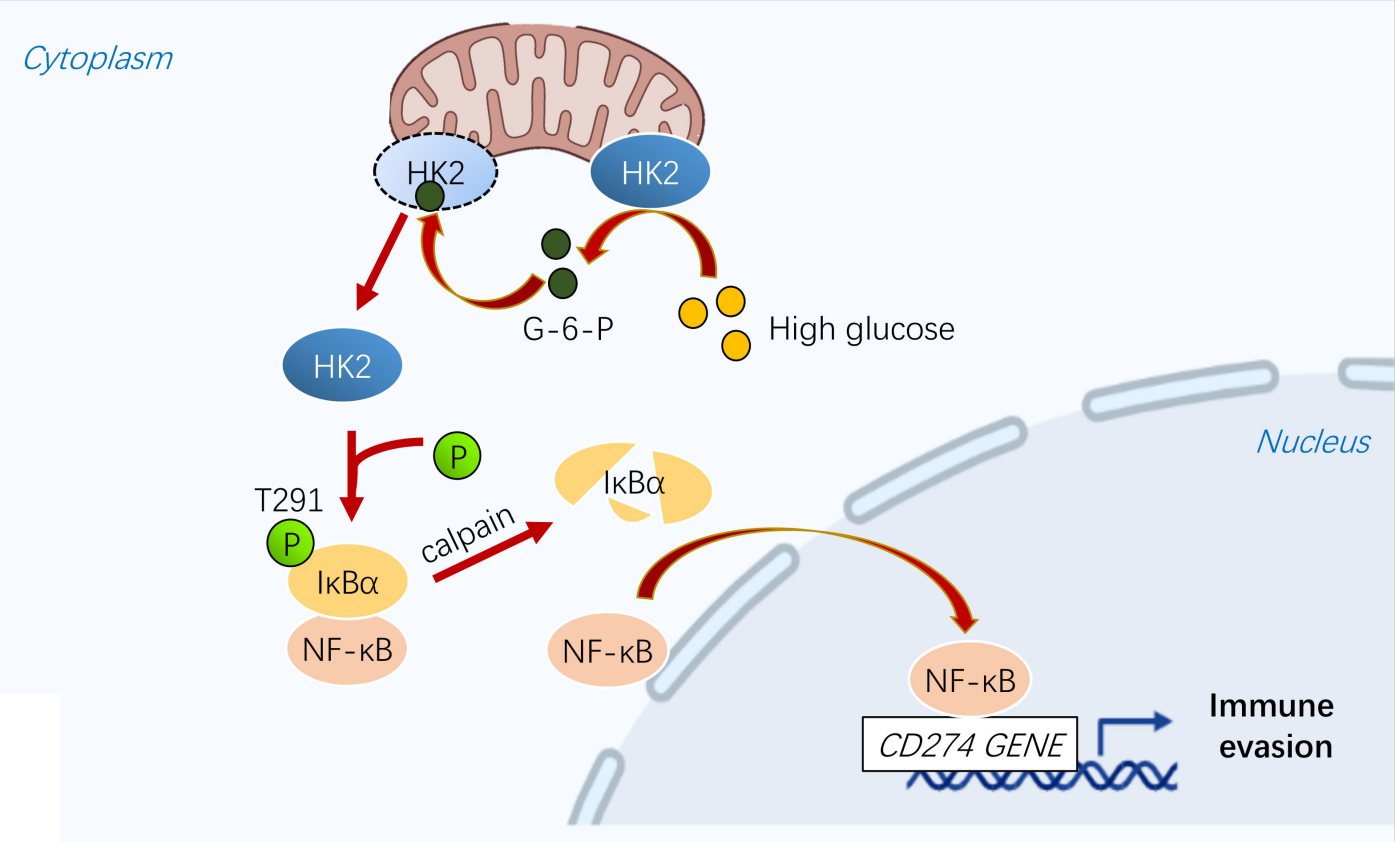
A schematic diagram shows that glycolytic enzyme HK2 acts as a protein kinase and phosphorylates IκBα to promote PD-L1 expression.

Metabolic reprogramming and immune evasion are characteristic of many cancers (41). PD-L1 is overexpressed in various tumors, including breast cancer, leading to immune evasion (42). PD-L1 can be regulated by different mechanisms. A recent study showed that energy deprivation activates AMPK kinase, which phosphorylates and promotes PD-L1 degradation (7, 43). Our study showed that high glucose regulates the transcription of PD-L1 in a NF-κB-dependent manner. In breast cancer cells, HK2 is highly expressed and is associated with the occurrence and progression of breast cancer (3, 44, 45). We demonstrated that HK2 plays a key role in regulating PD-L1 in breast cancer cells in response to high glucose (**Figure 6**).

Cell metabolism and gene expression are two fundamental biological processes that can be mutually regulated (27). Recent research demonstrated that metabolic enzymes could possess protein kinase activity to phosphorylate protein substrates (46). For instance, phosphoenolpyruvate carboxykinase1 (PCK1) (29), phosphoglycerate kinase 1 (PGK1) (47–49), ketohexokinase (KHK)-A (50, 51), pyruvate kinase M2 isoform (PKM2) (52–54), choline kinase α (CHKα) (55, 56) phosphorylate a variety of protein substrates thereby regulating instrumental cellular activities, such as gene expression. Intriguingly, it was shown that fructose-1,6-bisphosphatase 1 (FBP1) functions as a protein phosphatase to dephosphorylate histone H3, highlighting the critical control of protein phosphorylation and dephosphorylation by metabolic enzymes (57, 58). We showed here that HK2, acting as a protein kinase, phosphorylates IκBα at T291 in breast cancer cells, leading to IκBα degradation and subsequent activation of NF-κB for upregulation of PD-L1 transcription. Bioinformatic analysis showed that HK2 expression is associated with upregulated CD274 mRNA expression, reduced infiltration of CD4^+^ and CD8^+^ T cells in breast cancer specimens, and decreased survival time of breast cancer patients. In addition, the clinical significance of HK2-upregulated PD-L1 expression is evidenced by the positive correlation of HK2 with IκBα T291 phosphorylation and PD-L1 expression in human breast cancer samples. Our findings highlight the interplay between metabolic enzymes and tumor immunity, suggesting that HK2 serves as an effective molecular biomarker for PD-L1 antibody therapy.

## Data availability statement

The original contributions presented in the study are included in the article/**Supplementary Material**. Further inquiries can be directed to the corresponding authors.

## Ethics statement

The studies involving human participants were reviewed and approved by The institutional research ethics committee of the Oncology Department, Shandong Second Provincial General Hospital. Written informed consent for participation was not required for this study in accordance with the national legislation and the institutional requirements.

## Author contributions

LM, ZL, QiaW, and JLiu conceived and designed the study; JLin, WF, ZX, QiaW, HC and SC performed the experiments; JLin, WF, QiaW performed the statistical analysis and wrote the original draft. ZL and LM contributed to the final draft. All authors contributed to the article and approved the submitted version.

## Supplementary material

The Supplementary Material for this article can be foundonline at: https://www.frontiersin.org/articles/10.3389/fimmu.2023.1189953/full#supplementary-material
