# Supplementary material for: Glycolytic enzyme HK2 promotes PD-L1 expression and breast cancer cell immune evasion
Source: Front Immunol. 2023 Jun 12;14:1189953. doi: 10.3389/fimmu.2023.1189953 (PMC10291184; doi:10.3389/fimmu.2023.1189953)
Supplement: Supplementary file 1 [file DataSheet_1.pdf]

Figure 1

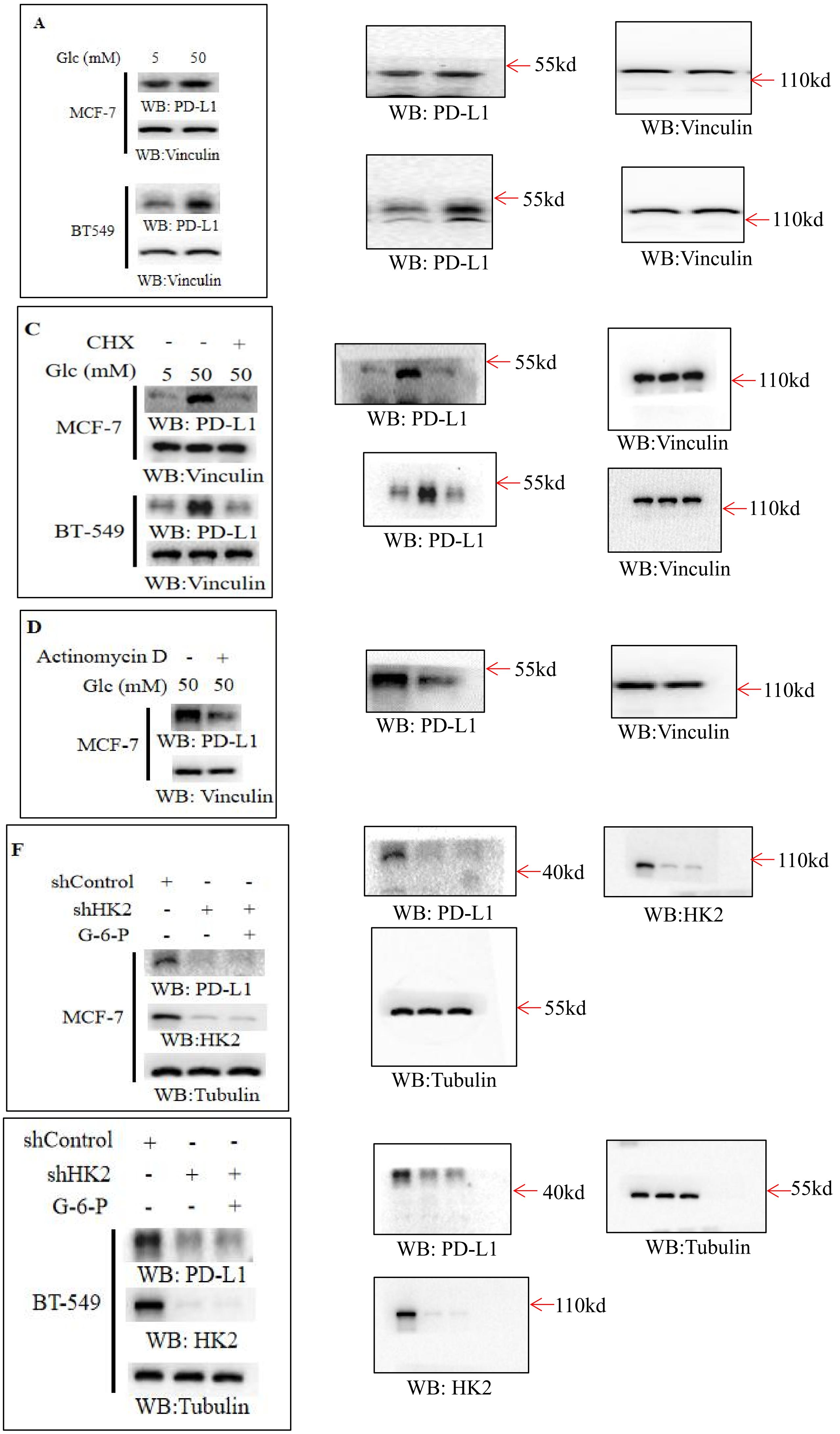
A
Glc (mM) 5 50
MCF-7
WB: PD-L1
WB:Vinculin
BT549
WB: PD-L1
WB:Vinculin
55kd
WB: PD-L1
110kd
WB:Vinculin
55kd
WB: PD-L1
110kd
WB:Vinculin
C
CHX - - +
Glc (mM) 5 50 50
MCF-7
WB: PD-L1
WB:Vinculin
BT-549
WB: PD-L1
WB:Vinculin
55kd
WB: PD-L1
110kd
WB:Vinculin
55kd
WB: PD-L1
110kd
WB:Vinculin
D
Actinomycin D - +
Glc (mM) 50 50
MCF-7
WB: PD-L1
WB: Vinculin
55kd
WB: PD-L1
110kd
WB:Vinculin
F
shControl + - -
shHK2 - + +
G-6-P - - +
MCF-7
WB: PD-L1
WB:HK2
WB:Tubulin
40kd
WB: PD-L1
110kd
WB:HK2
55kd
WB:Tubulin
shControl + - -
shHK2 - + +
G-6-P - - +
BT-549
WB: PD-L1
WB: HK2
WB:Tubulin
40kd
WB: PD-L1
55kd
WB:Tubulin
110kd
WB: HK2

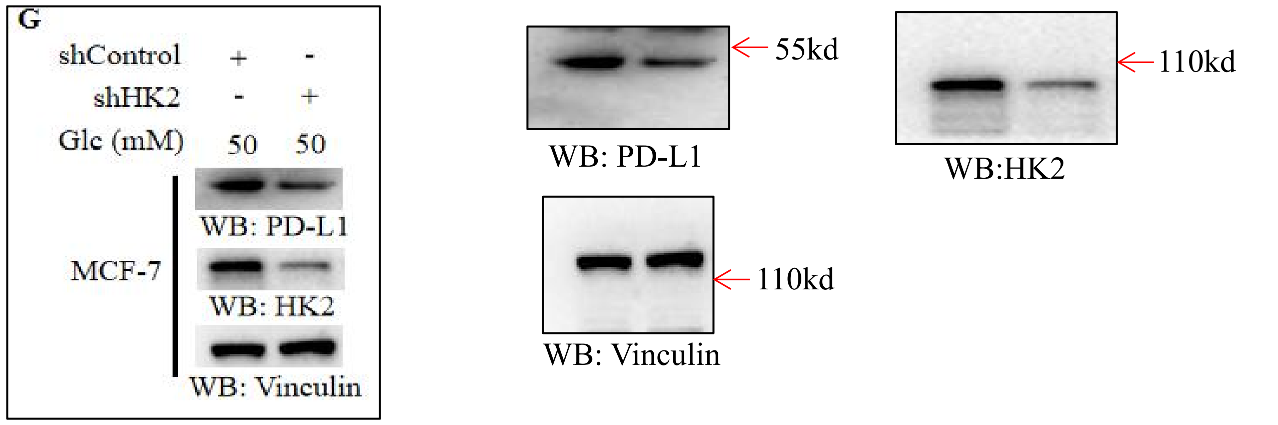
G
shControl + -
shHK2 - +
Glc (mM) 50 50
MCF-7
WB: PD-L1
WB: HK2
WB: Vinculin
55kd
WB: PD-L1
110kd
WB:HK2
110kd
WB: Vinculin

Figure 2

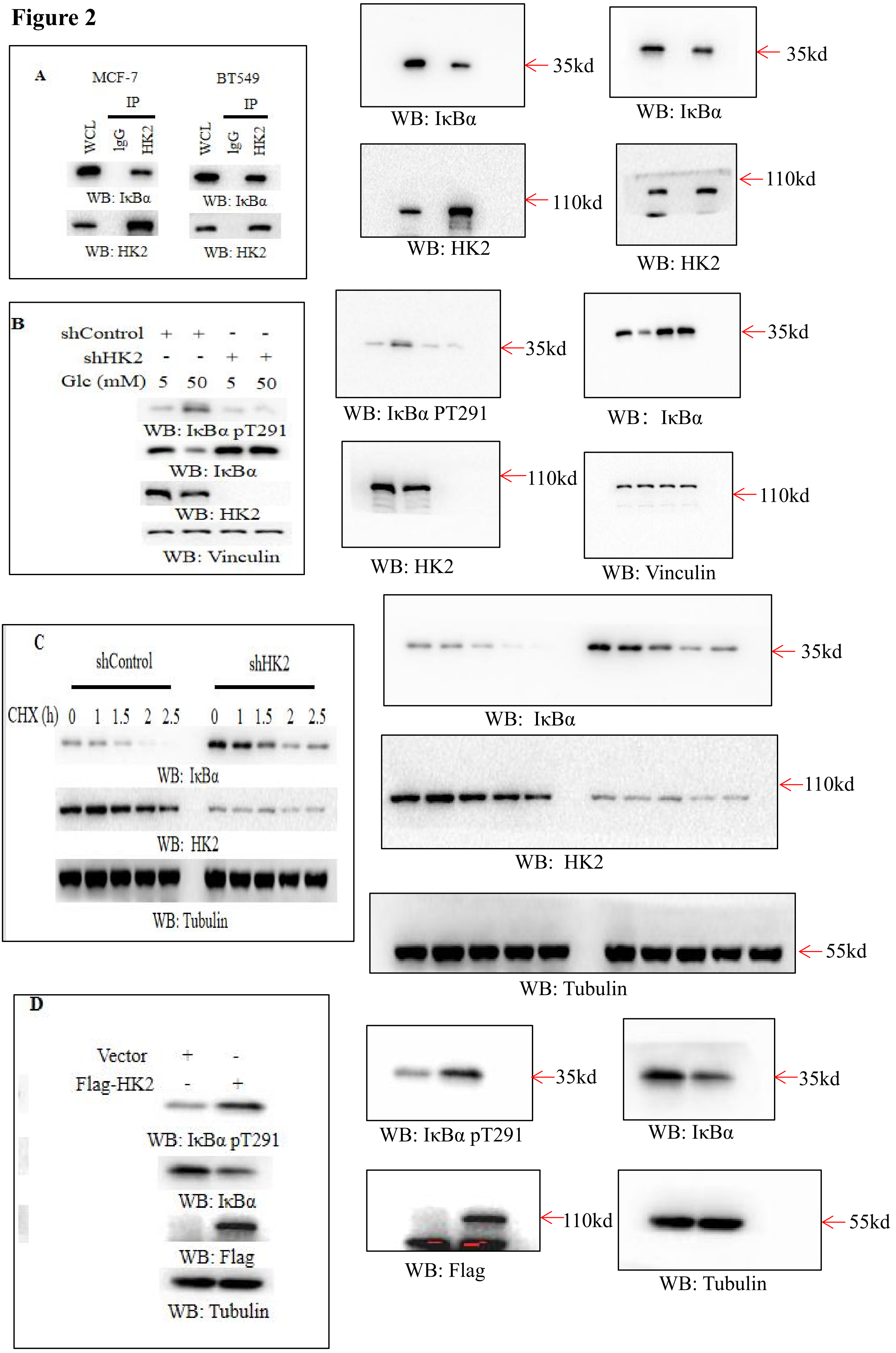
A
MCF-7
BT549
IP
WCL
IgG
HK2
WB: IκBα
WB: HK2
35kd
WB: IκBα
110kd
WB: HK2
B
shControl + + - -
shHK2 - - + +
Glc (mM) 5 50 5 50
WB: IκBα pT291
WB: IκBα
WB: HK2
WB: Vinculin
35kd
WB: IκBα PT291
110kd
WB: Vinculin
C
shControl
shHK2
CHX (h) 0 1 1.5 2 2.5 0 1 1.5 2 2.5
WB: IκBα
WB: HK2
WB: Tubulin
55kd
D
Vector + -
Flag-HK2 - +
WB: IκBα pT291
WB: IκBα
WB: Flag
WB: Tubulin

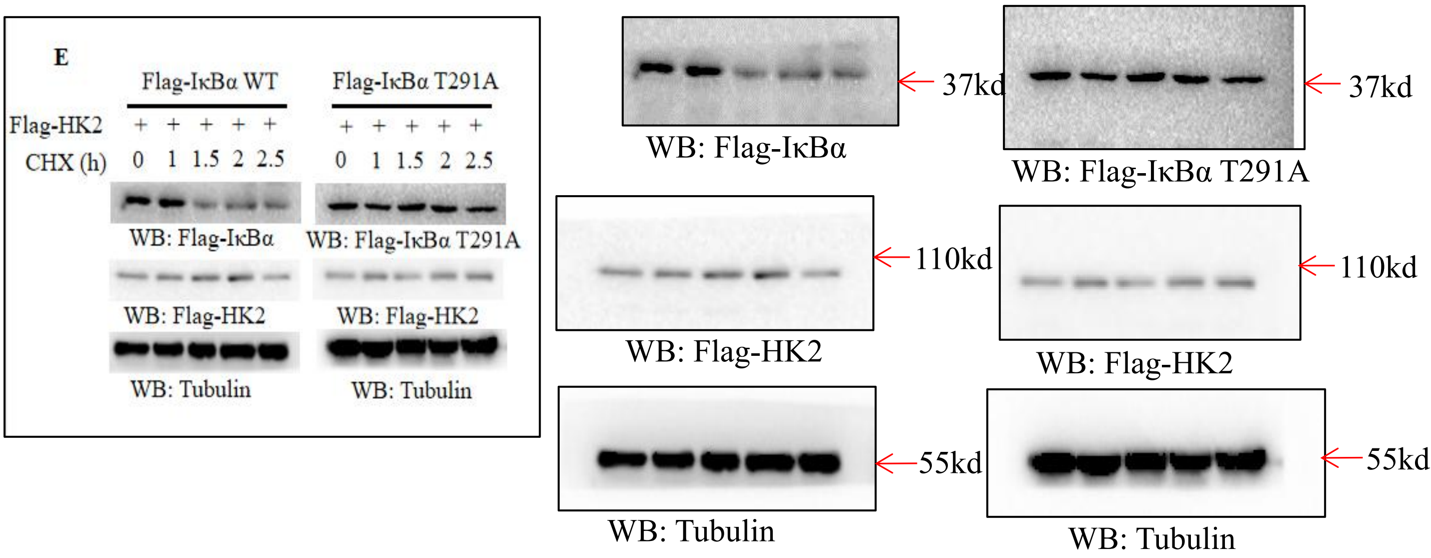
E
Flag-IκBα WT
Flag-IκBα T291A
Flag-HK2 + + + + + + + + + +
CHX (h) 0 1 1.5 2 2.5 0 1 1.5 2 2.5
WB: Flag-IκBα
WB: Flag-IκBα T291A
WB: Flag-HK2
WB: Flag-HK2
WB: Tubulin
WB: Tubulin
37kd
WB: Flag-IκBα
37kd
WB: Flag-IκBα T291A
110kd
WB: Flag-HK2
110kd
WB: Flag-HK2
55kd
WB: Tubulin
55kd
WB: Tubulin

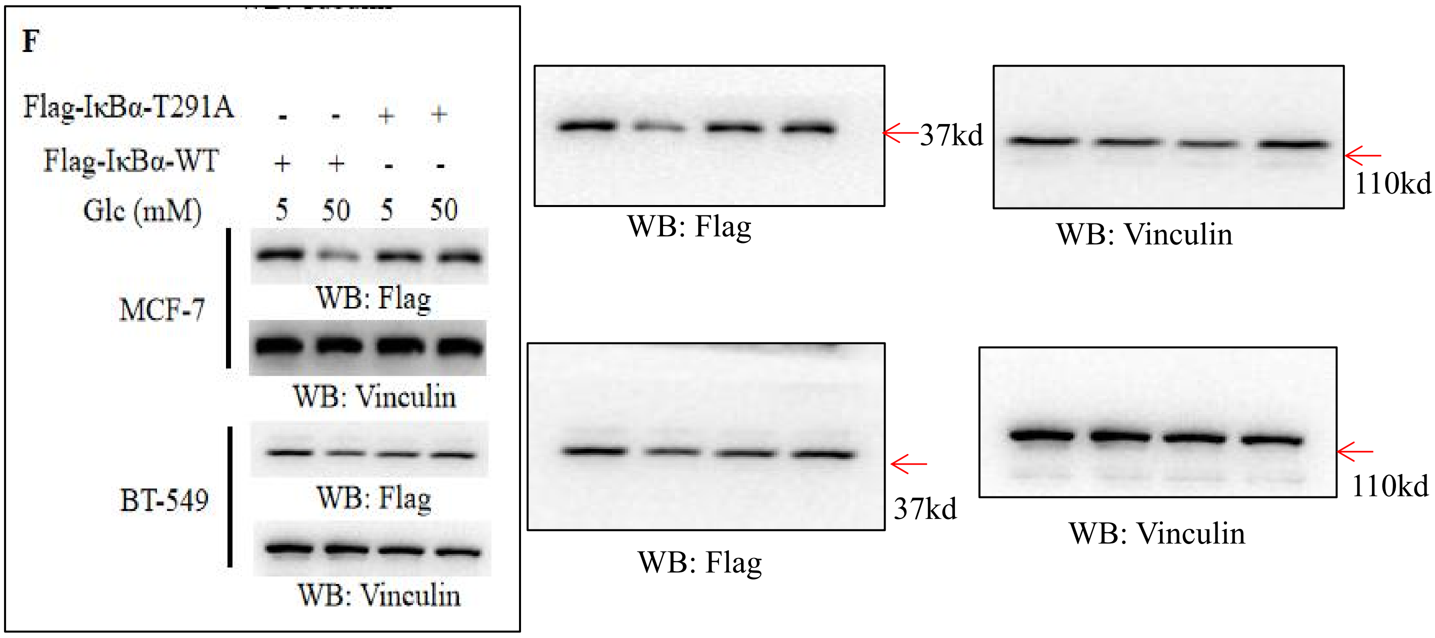
F
Flag-IκBα-T291A - - + +
Flag-IκBα-WT + + - -
Glc (mM) 5 50 5 50
MCF-7
WB: Flag
WB: Vinculin
BT-549
WB: Flag
WB: Vinculin
37kd
WB: Flag
110kd
WB: Vinculin
37kd
WB: Flag
110kd
WB: Vinculin

Figure 3

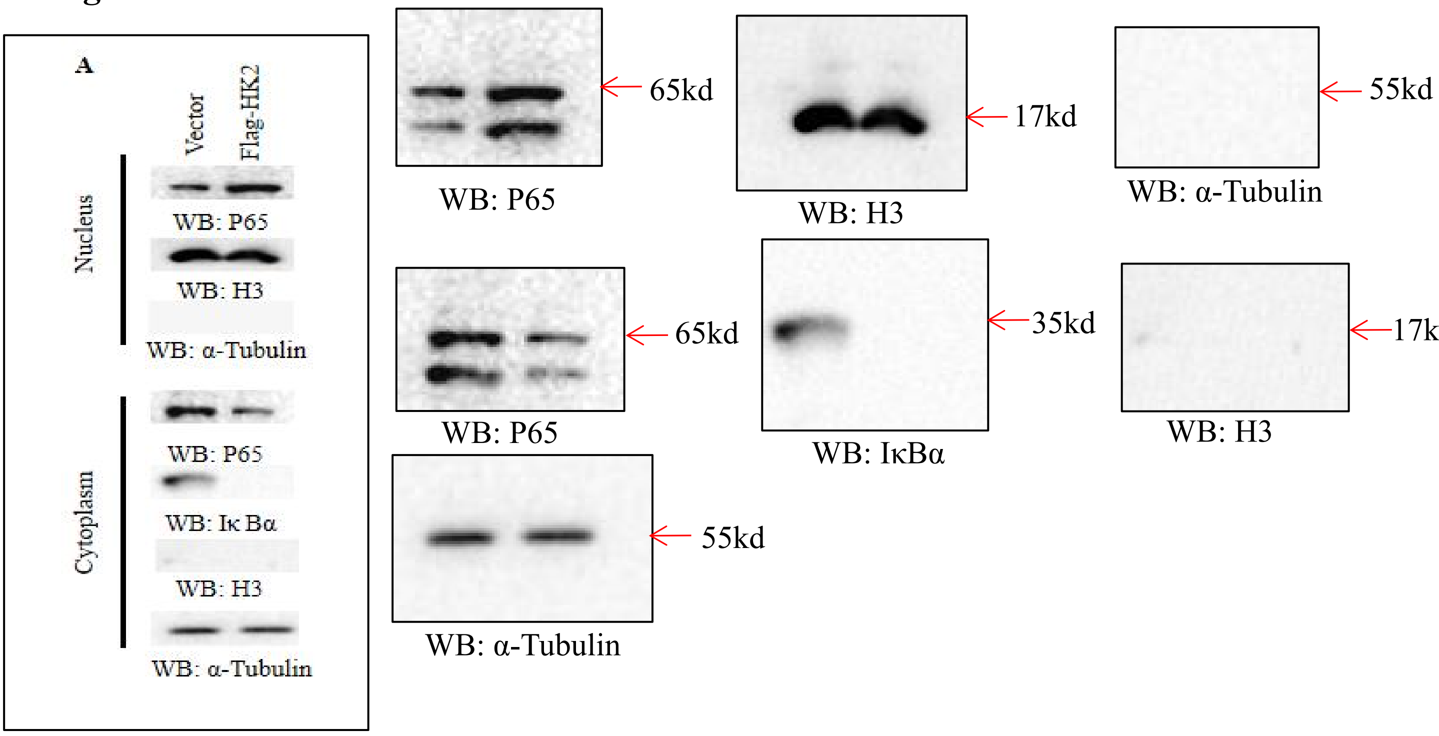
A
Vector
Flag-HK2
Nucleus
WB: P65
WB: H3
WB: α-Tubulin
Cytoplasm
WB: P65
WB: Iκ Bα
WB: H3
WB: α-Tubulin
65kd
WB: P65
17kd
WB: H3
55kd
WB: α-Tubulin
65kd
WB: P65
35kd
WB: IκBα
17k
WB: H3
55kd
WB: α-Tubulin

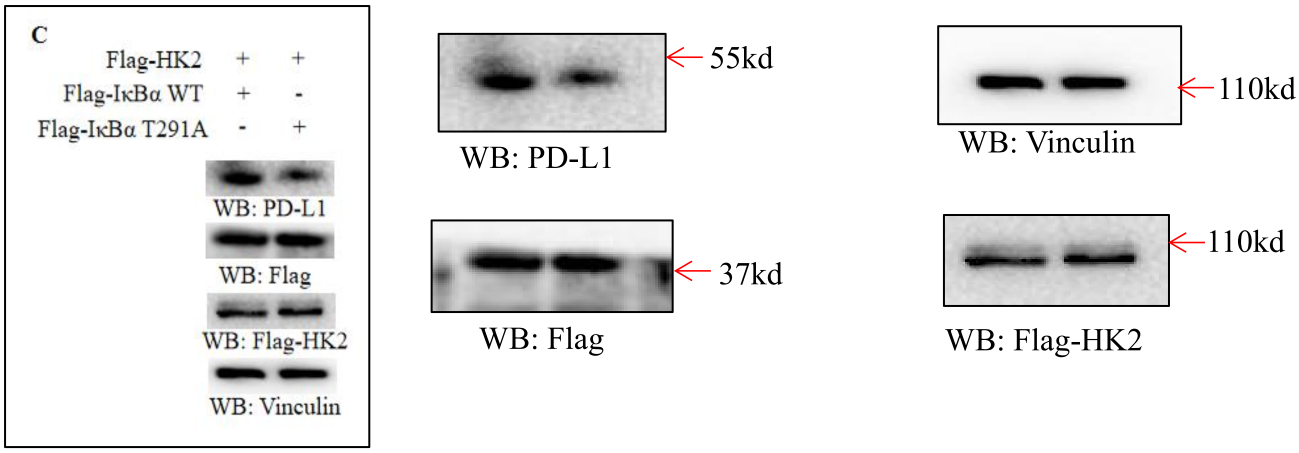
C
Flag-HK2 + +
Flag-IκBα WT + -
Flag-IκBα T291A - +
WB: PD-L1
WB: Flag
WB: Flag-HK2
WB: Vinculin
55kd
WB: PD-L1
110kd
WB: Vinculin
37kd
WB: Flag
110kd
WB: Flag-HK2

D

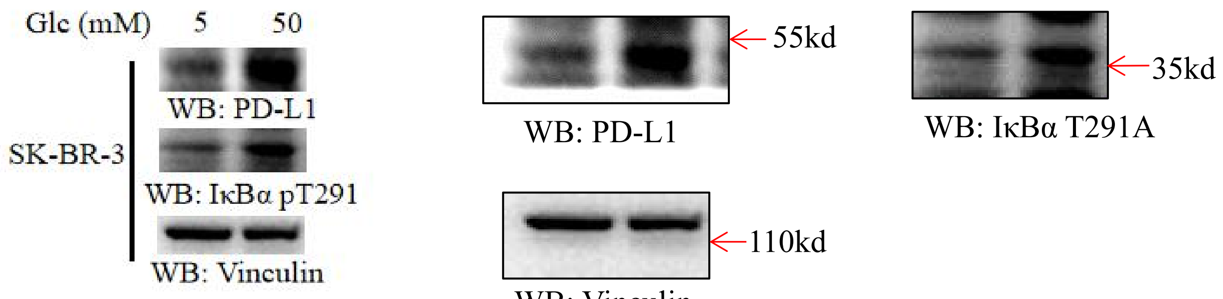

Figure 4

A

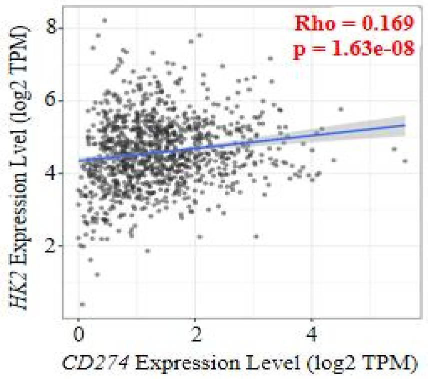

A. TIMER2.0 (http://timer.cistrome.org/)
Total 1100 breast cancer cases in TIMER2.0 database based on the Cancer Genome Atlas (TCGA) dataset(1-4).

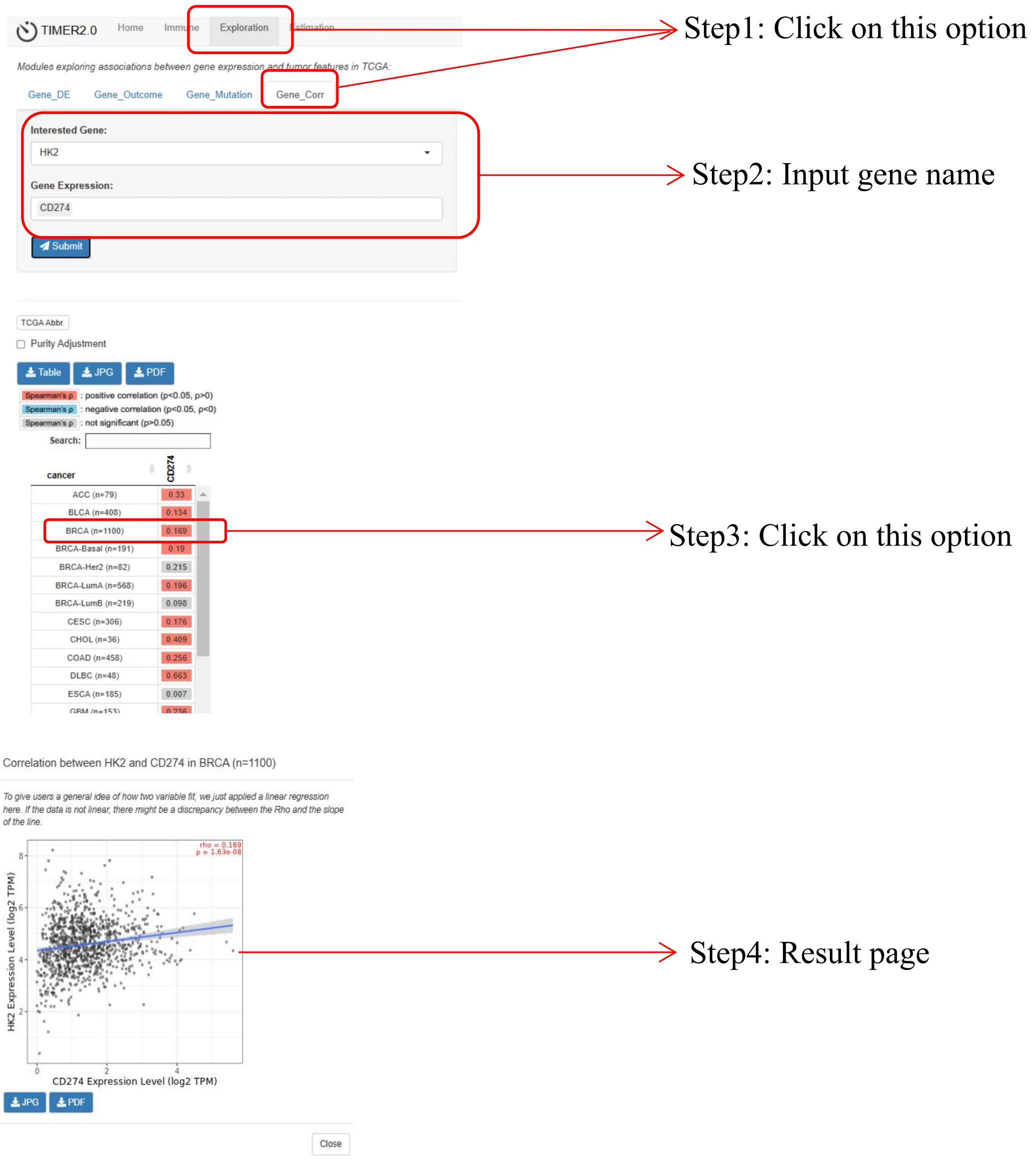

References
1. Li T, Fu J, Zeng Z, Cohen D, Li J, Chen Q, et al. TIMER2.0 for analysis of tumor-infiltrating immune cells. Nucleic Acids Res. 2020;48(W1):W509-w14.
2. Li T, Fan J, Wang B, Traugh N, Chen Q, Liu JS, et al. TIMER: A Web Server for Comprehensive Analysis of Tumor-Infiltrating Immune Cells. Cancer Res. 2017;77(21):e108-e10.
3. Li B, Severson E, Pignon JC, Zhao H, Li T, Novak J, et al. Comprehensive analyses of tumor immunity: implications for cancer immunotherapy. Genome Biol. 2016;17(1):174.
4. Weinstein JN, Collisson EA, Mills GB, Shaw KR, Ozenberger BA, Ellrott K, et al. The Cancer Genome Atlas Pan-Cancer analysis project. Nat Genet. 2013;45(10):1113-20.

Figure 4

B

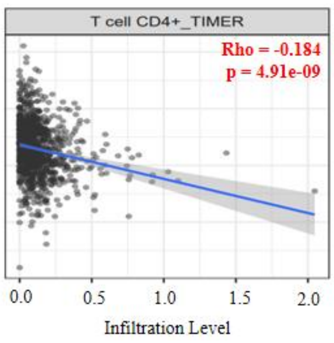

B. TIMER2.0 (http://timer.cistrome.org/)
TIMER algorithm was applied to the expression profiles of the Cancer Genome Atlas (TCGA) tumors to analyze a sample size of 1100 breast cancer cases(1-4).

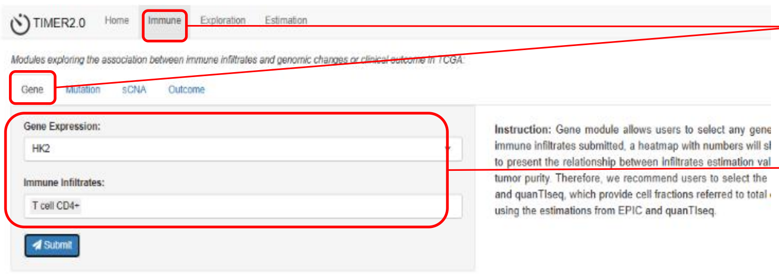

Step1: Click on this option

Step2: Input gene name

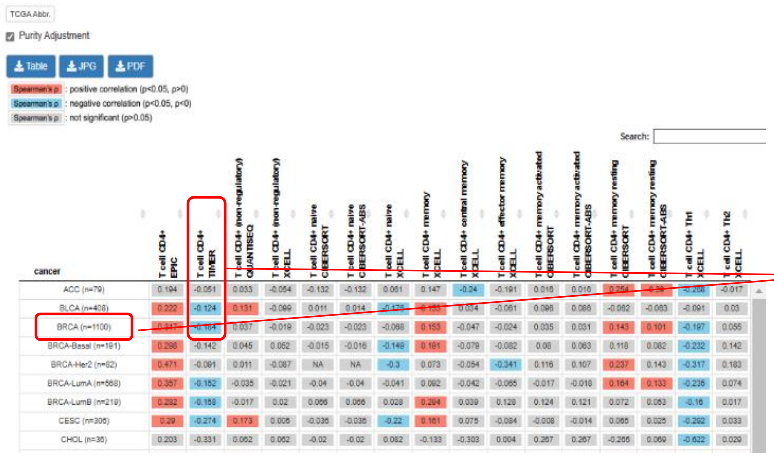

Step3: Click on this option

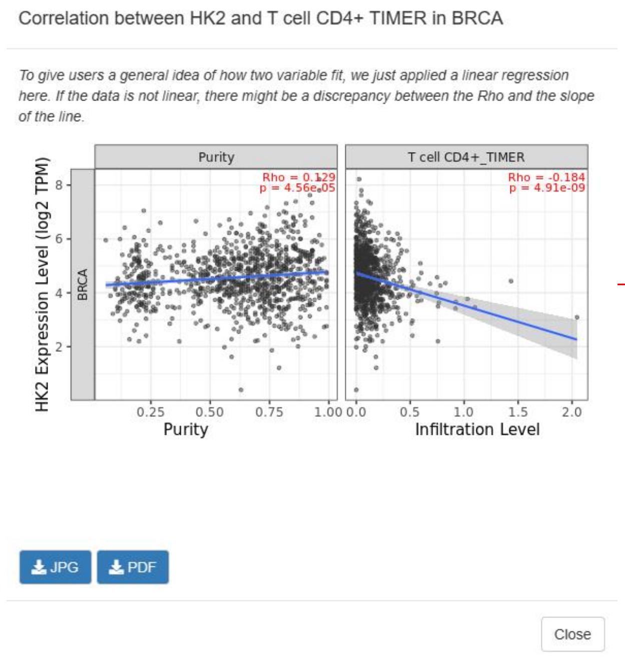

Step4: Result page

References

1. Li T, Fu J, Zeng Z, Cohen D, Li J, Chen Q, et al. TIMER2.0 for analysis of tumor-infiltrating immune cells. Nucleic Acids Res. 2020;48(W1):W509-w14.
2. Li T, Fan J, Wang B, Traugh N, Chen Q, Liu JS, et al. TIMER: A Web Server for Comprehensive Analysis of Tumor-Infiltrating Immune Cells. Cancer Res. 2017;77(21):e108-e10.
3. Li B, Severson E, Pignon JC, Zhao H, Li T, Novak J, et al. Comprehensive analyses of tumor immunity: implications for cancer immunotherapy. Genome Biol. 2016;17(1):174.
4. Weinstein JN, Collisson EA, Mills GB, Shaw KR, Ozenberger BA, Ellrott K, et al. The Cancer Genome Atlas Pan-Cancer analysis project. Nat Genet. 2013;45(10):1113-20.

Figure 4

C

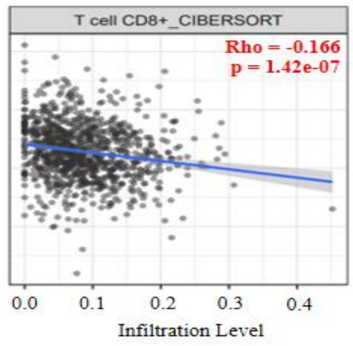

C. TIMER2.0 （http://timer.cistrome.org/)
CIBERSORT algorithm was applied to the expression profiles of the Cancer Genome Atlas (TCGA) tumors to analyze a sample size of 1100 breast cancer cases(1-4).

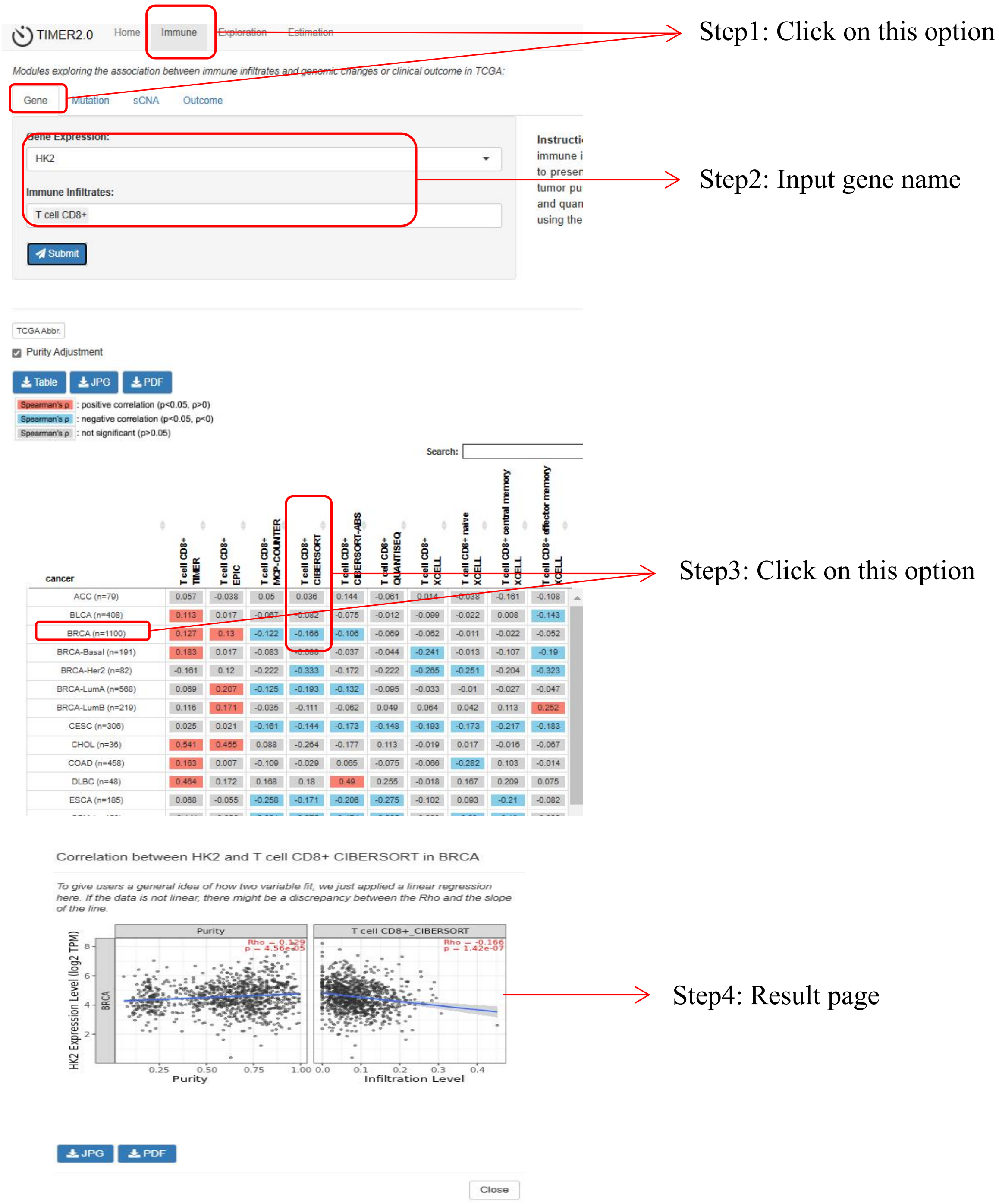

References
1. Li T, Fu J, Zeng Z, Cohen D, Li J, Chen Q, et al. TIMER2.0 for analysis of tumor-infiltrating immune cells. Nucleic Acids Res. 2020;48(W1):W509-w14.
2. Li T, Fan J, Wang B, Traugh N, Chen Q, Liu JS, et al. TIMER: A Web Server for Comprehensive Analysis of Tumor-Infiltrating Immune Cells. Cancer Res. 2017;77(21):e108-e10.
3. Li B, Severson E, Pignon JC, Zhao H, Li T, Novak J, et al. Comprehensive analyses of tumor immunity: implications for cancer immunotherapy. Genome Biol. 2016;17(1):174.
4. Weinstein JN, Collisson EA, Mills GB, Shaw KR, Ozenberger BA, Ellrott K, et al. The Cancer Genome Atlas Pan-Cancer analysis project. Nat Genet. 2013;45(10):1113-20.

Figure4 D

D

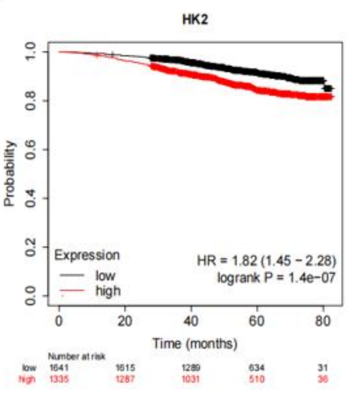

D Kaplan Meier plotter (https://kmplot.com/analysis/)
A breast cancer cohort containing 2976 breast cancer cases in NCBI Gene Expression Omnibus (https://www.ncbi.nlm.nih.gov/geo/) and in the Genomic Data Commons Data Portal (https://portal.gdc.cancer.gov/) (5,6).

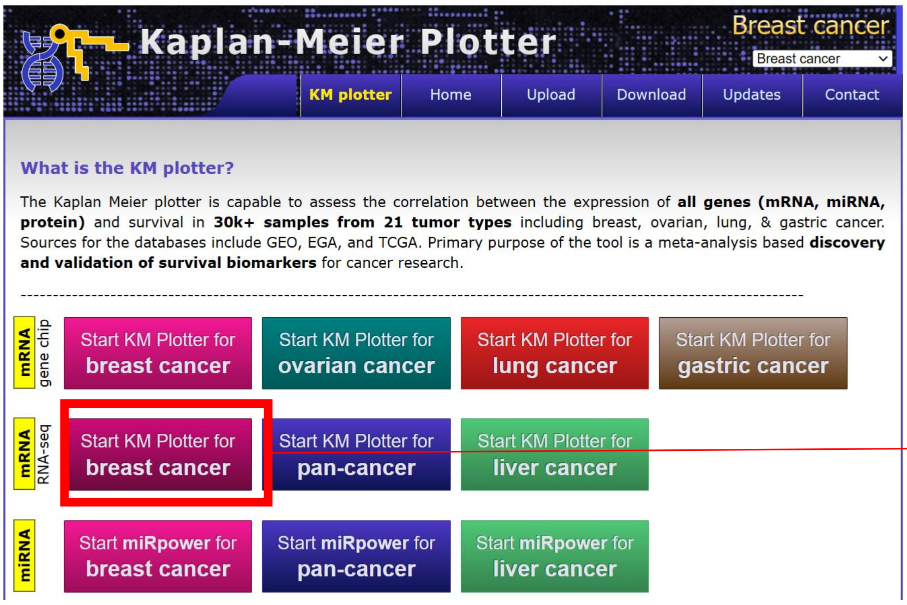

Step1: Click on this option

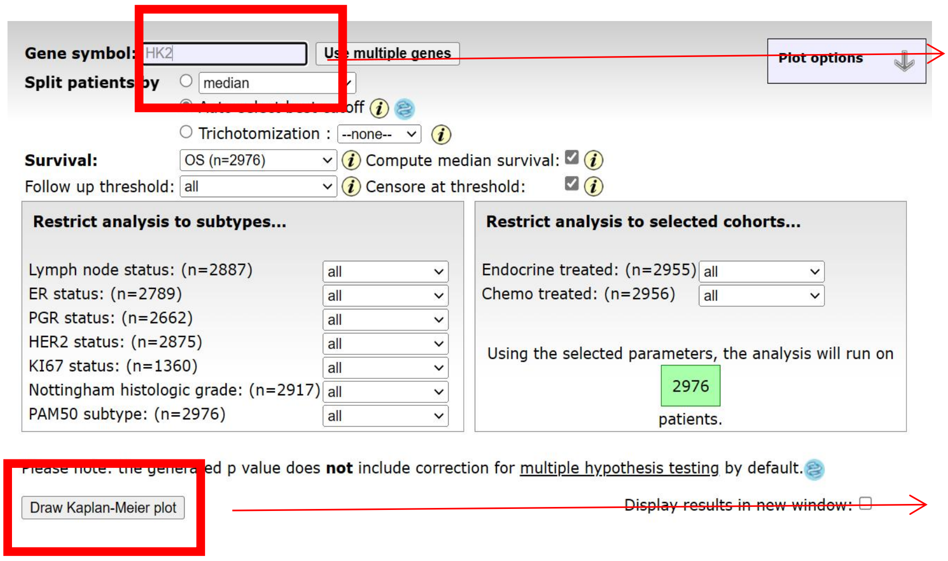

Step2: Input gene name

Step3: Draw plot

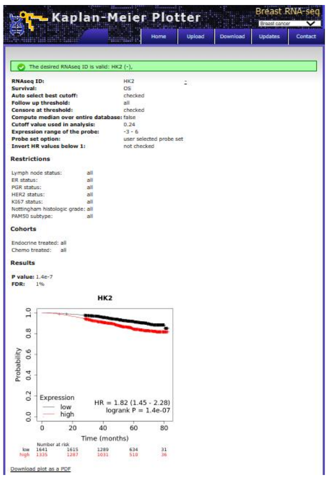

Step4：Result page

5. Győrffy B. Discovery and ranking of the most robust prognostic biomarkers in serous ovarian cancer. Geroscience. 2023.
6. Lánczky A, Győrffy B. Web-Based Survival Analysis Tool Tailored for Medical Research (KMplot): Development and Implementation. J Med Internet Res. 2021;23(7):e27633.

Figure 5

A

BRCA

HK2 | IκBα pT291 | PD-L1

Tumor 1

Tumor 2

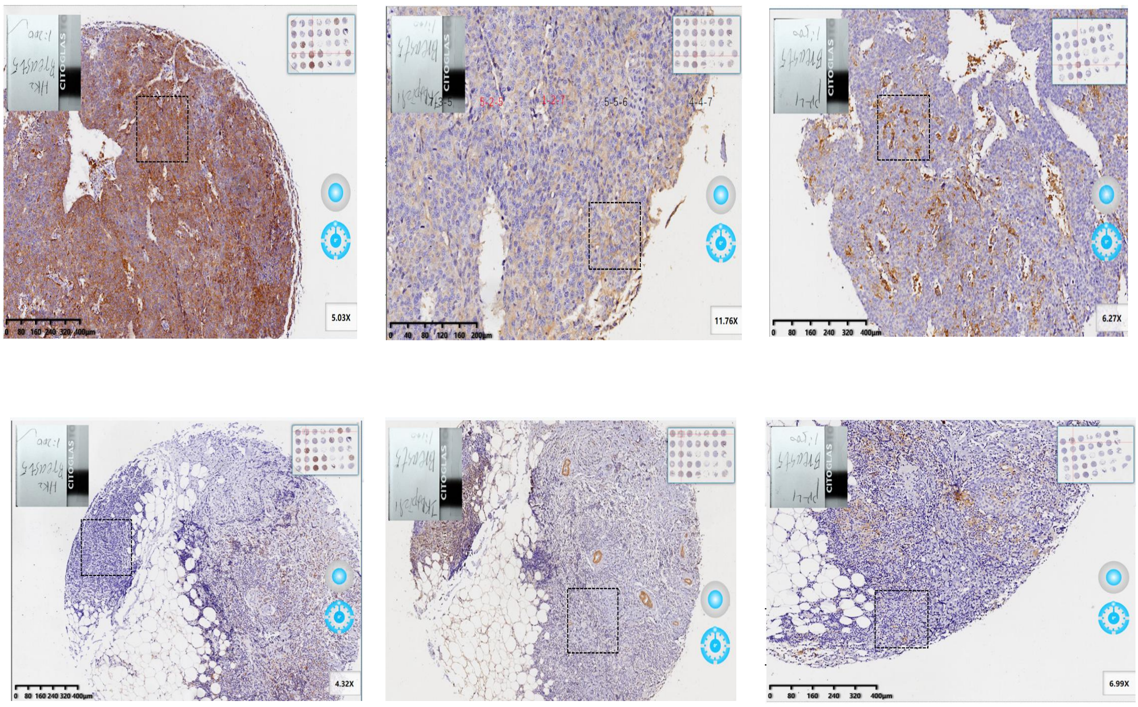

B

Clinical data analysis is presented in Excel 1.
